# Supplementary material for: High-throughput fabrication of soft magneto-origami machines
Source: Nat Commun. 2022 Jul 19;13:4177. doi: 10.1038/s41467-022-31900-5 (PMC9296529; doi:10.1038/s41467-022-31900-5)
Supplement: Supplementary file 1 — Supplementary Information [file 41467_2022_31900_MOESM1_ESM.pdf]

Supplementary Materials for

High-throughput Fabrication of Soft Magneto-origami Machines

Shengzhu Yi,[1]† Liu Wang,[2] † Zhipeng Chen,[1]† Jian Wang,[1] Xingyi Song,[1] Pengfei Liu,[1] Yuanxi Zhang,[1] Qingqing Luo,[1] Lelun Peng,[1] Zhigang Wu[3,*], Chuan Fei Guo,[4,5,6]* Lelun Jiang [1, *]

1. Guangdong Provincial Key Laboratory of Sensor Technology and Biomedical Instrument, School of Biomedical Engineering, Sun Yat-Sen University, Shenzhen 518107, China.
2. CAS Key Laboratory of Mechanical Behavior and Design of Materials, Department of Modern Mechanics, University of Science and Technology of China, Hefei 230026, China
3. State Key Laboratory of Digital Manufacturing Equipment and Technology, Huazhong University of Science and Technology, Wuhan 430074, China
4. Department of Materials Science and Engineering, Southern University of Science and Technology, Shenzhen 518055, China.
5. Shenzhen Engineering Research Center for Novel Electronic Information Materials and Devices, Southern University of Science and Technology, 518055 Shenzhen, China.
6. Guangdong Provincial Key Laboratory of Functional Oxide Materials and Devices, Southern University of Science and Technology, Shenzhen 518055, China.

*Corresponding authors.
E-mail: zgwu@hust.edu.cn (Z. Wu); guocf@sustech.edu.cn (C.F.G); jianglel@mail.sysu.edu.cn (L. Jiang).

† These authors contributed equally to this work.

The PDF file includes

Supplementary Table 1

Supplementary Figures 1-21

Supplementary Table 1. Comparison between this work and existing soft magneto-active machines.

Fabrication Method	Materials	Magnetization encoding	Structural complexity	Time cost	Production scale	Locomotion modes	Reference
3D printing	Hard magnetic particle @ polymer matrix	Sequential encoding while printing	Low	Intermediate	Intermediate	Bending/rolling /walking	(13-15)
Laser printing	Hard magnetic particle @ polymer matrix	Sequential encoding while heating	Low	High	Small	Bending/rolling /walking	(16-17)
2D template molding	Soft magnetic particle @ polymer matrix	Encoding at one time while curing	Low	High	Intermediate	Bending/Crawling	(12)
Voxel assembly	NdFeB @ PDMS voxel +Dragon skin voxel +Ecoflex bonding agent	Encoding before assembly	High	High	Small	Bending/twisting /contraction	(18)
Transfer printing	NdFeB @ PDMS +Stamp film +Adhesive	Encoding before assembly	Intermediate	High	Small	Bending/crawling	(19)
Origami +Assembly	Permanent magnets +Origami (e.g, polystyrene)	Permanent magnet orientation	High	Low	Intermediate	Contraction/deploying /bending/twisting	(29-33)
Origami +Magnetic Spray	Fe @ PVA&Gluten + Paper	Sequential encoding while curing	High	High	Small	Folding/rolling /walking/crawling	(35)
Origami	Magnetic sheet: NeFeB+ PEG shell @ Ecoflex	Encoding at one time while heating	Intermediate	High	Small	Bending/folding	(34)
**Origami + Roll-to-roll**	**Magnetic sheet: NeFeB @ Ecoflex on paper**	**Encoding at one time after curing**	**High**	**Low**	**Large**	**Bending/folding/ contraction/ deploying/rolling**	**This work**

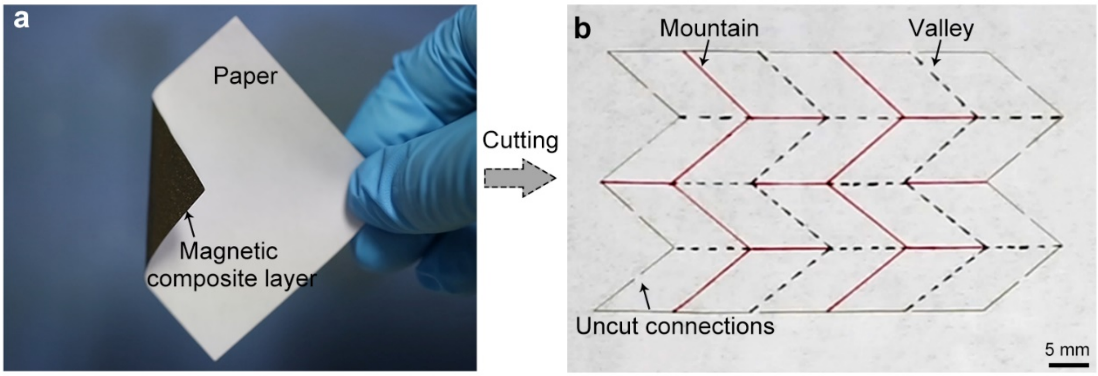

Supplementary Figure 1. Laser cutting and drawing of origami patterns on a magnetic sheet for tearing off and folding. (a) Optical image of a magnetic sheet. The magnetic paper consists of a paper layer and a magnetic composite layer. (b) Programmed magnetic paper with predesigned origami patterns and discontinuous cutting path. The origami pattern can be printed or drew on the paper layer, and a laser machine is used to cut the paper. The uncut connections enable easy tear-off of the 2D pattern. The laser output power is 5 w. The laser linewidth is 0.15 mm. The wavelength is 1064 nm. The environment temperature is 26 °C. The laser pulse width is 800 ns.

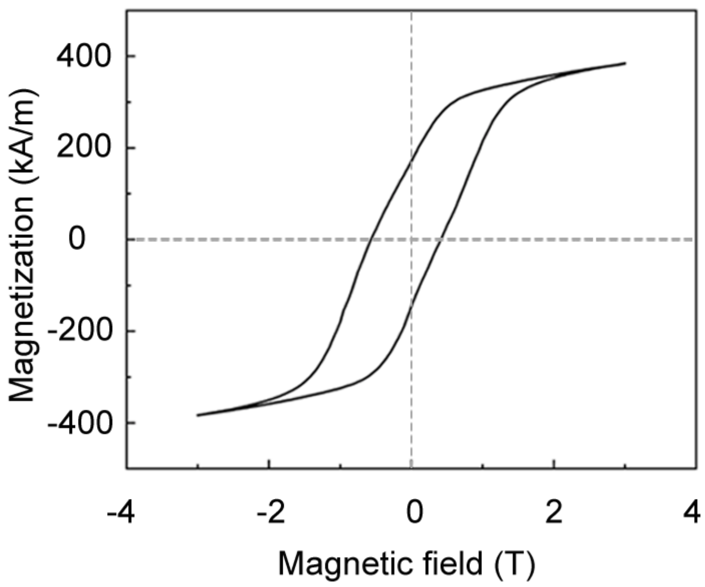

Supplementary Figure 2. The hysteresis loop of magnetic composite. The residual magnetization and the relative permeability of magnetic paper are 170 kA·m^{-1} and 1.05, respectively.

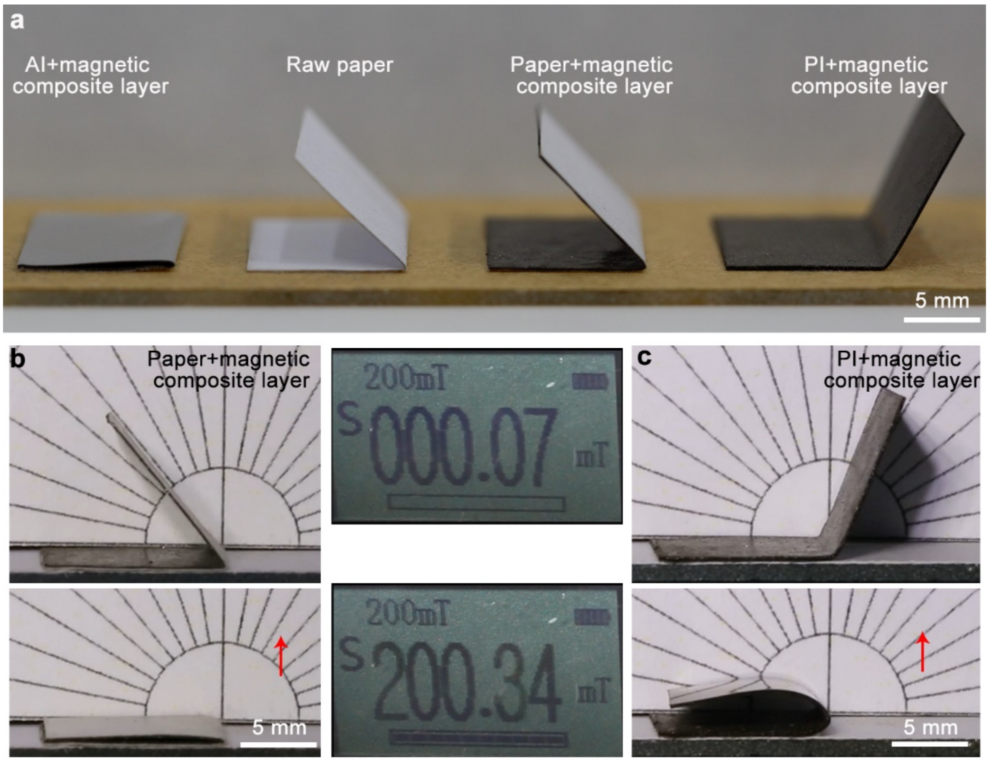

Supplementary Figure 3. Influence of different materials on fabrication and deformation of magneto-origami machines. (a) Magneto-origami machines using different materials, *e.g.*, aluminium (Al) film, paper, and polyimide (PI) film. Folding performance of magneto-origami machines fabricated through paper (b) and PI (c), under a 200 mT magnetic actuation.

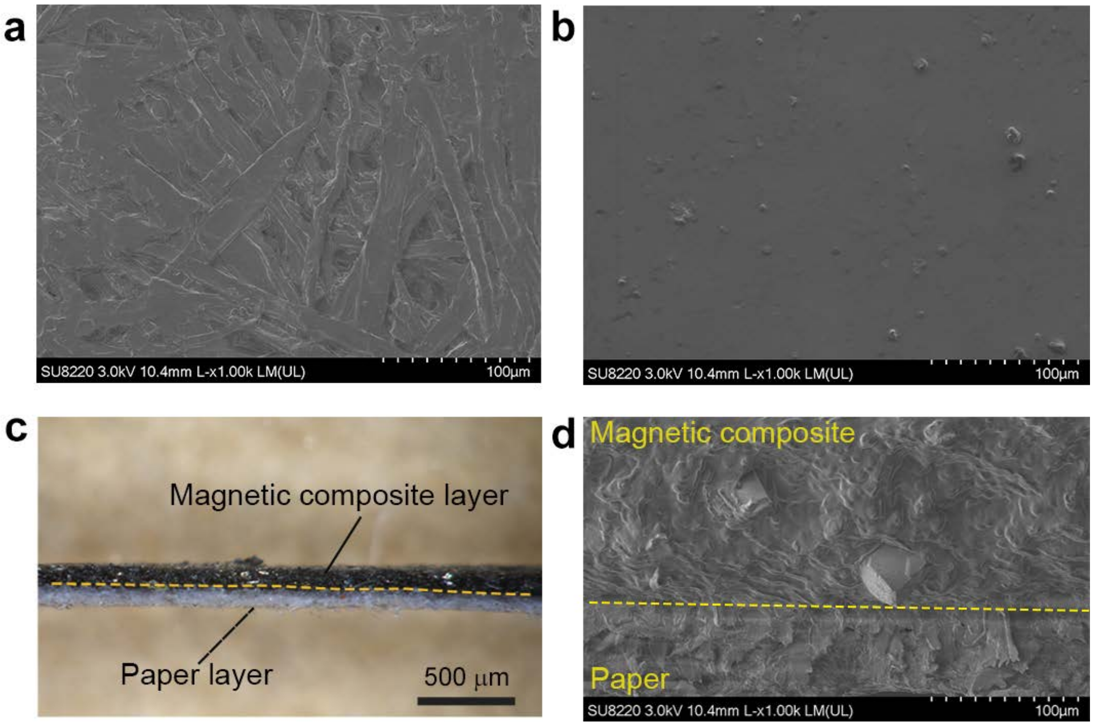

Supplementary Figure 4. Characterization of the magnetic sheet. Scanning electron microscope (SEM) images of (a) paper, and (b) magnetic composite layer. (c-d) Optical and SEM images of the cross-section of the magnetic sheet, respectively. The yellow dotted line represents the boundary between paper and the magnetic composite layer. The thickness of the paper and the magnetic composite layer is 90 µm and 100 µm, respectively.

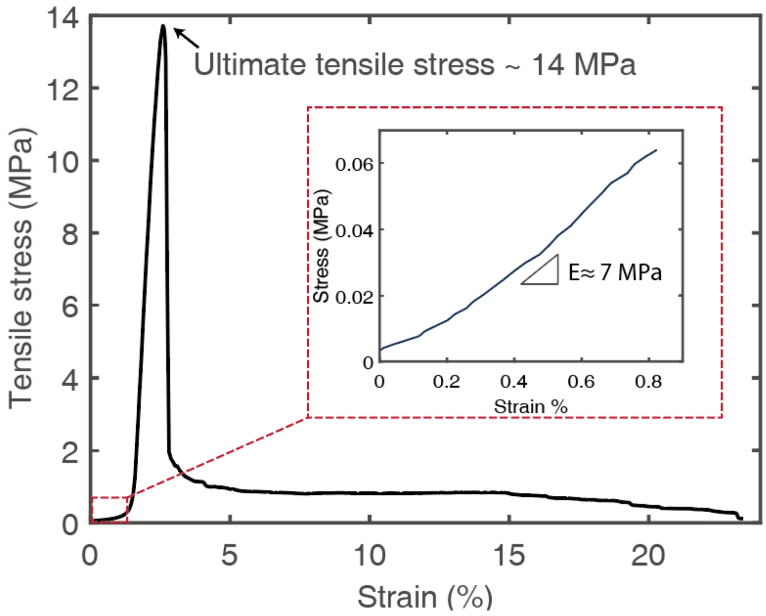

Supplementary Figure 5. Mechanical properties of the magnetic sheet. Young's modulus is 7 MPa and ultimate tensile stress is 14 MPa.

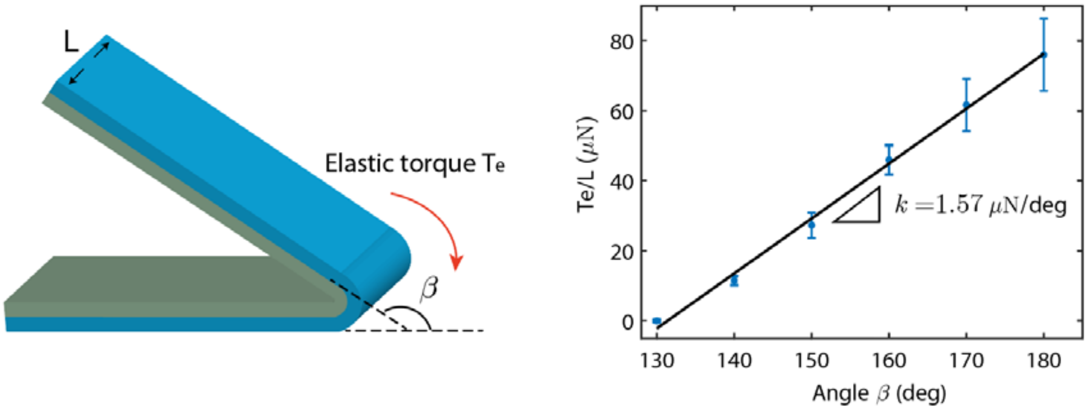

Supplementary Figure 6. Measurement of the elastic torque constant of the folding crease.

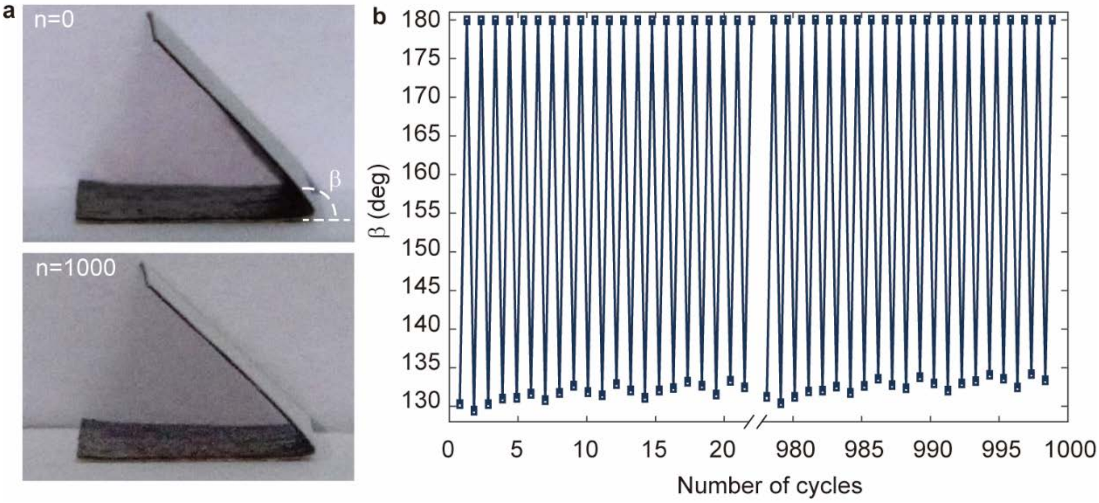

Supplementary Figure 7. A single-fold magneto-origami machine under cyclic magnetic actuation. (a) Images of the single-fold magneto-origami machine under different cycles. (b) The angle of the single-fold magneto-origami machine almost remains almost unchanged after 1000 cycles. The mean and standard deviation of the maximum angle are 180°and 0° (fully folded), respectively. The mean and standard deviation of the min angle are 131° and 1°, respectively.

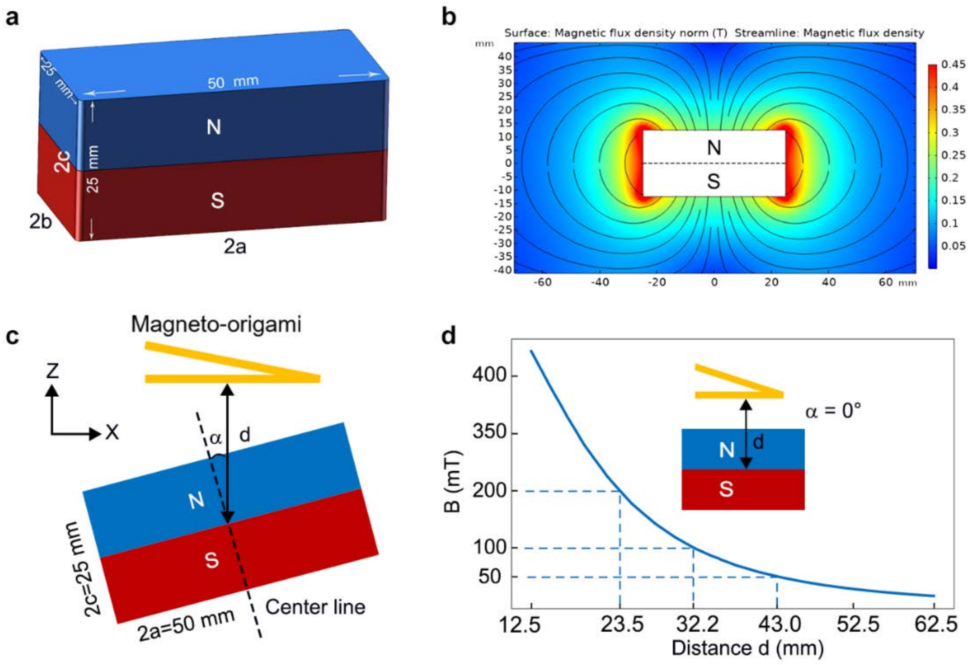

Supplementary Figure 8. A cuboid permanent magnet for actuating magneto-origami machines. (a) Schematic of the cuboid magnet with 25 mm in width, 50 mm in length, and 25 mm in height. (b) The magnetic field distribution around the cuboid magnet. The distribution and magnetization of the permanent magnet was obtained using COMSOL Multiphysics software under the same conditions including dimensions and magnetization. (c) Schematic of two parameters (distance *d* and angle *α*) to quantitatively control the permanent magnet. (d) The magnetic field strength as a function of distance *d* along the centerline of the magnet (*α*=0°).

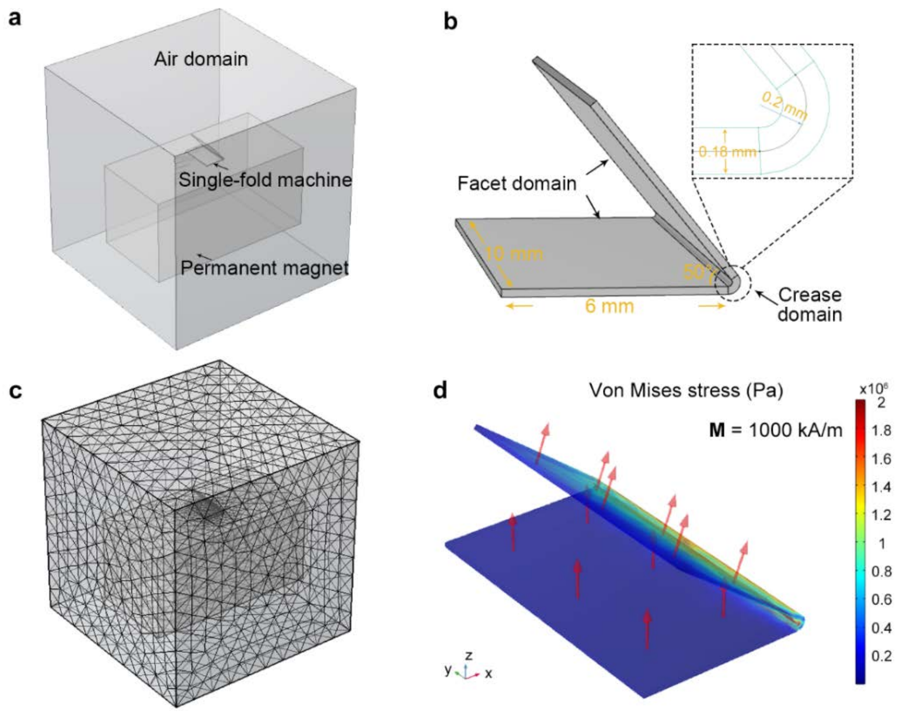

Supplementary Figure 9. Finite element analysis (FEA) of single-crease magneto-origami machine actuated by a cuboid permanent magnet. (a) FEA model of single-crease magneto-origami machines. (b) The FEA model for single-crease magneto-origami machine and detailed parameters of the model. (c) Meshed FEA model in COMSOL. (d) Folding state of the single-fold model when the residual magnetization the permanent magnet is 1000 $\mathrm{kA \cdot m^{-1}}$.

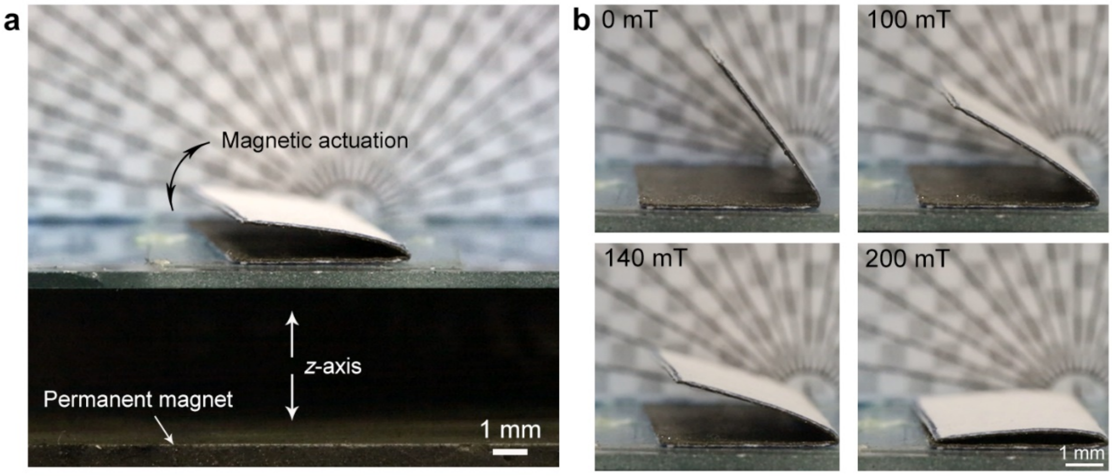

Supplementary Figure 10. Experimental demonstration of the single-crease magneto-origami machine. (a) Experimental setup for actuating the single-crease magneto-origami machine. (b) Folding behaviors of single-crease magneto-origami machine under different actuation magnetic fields.

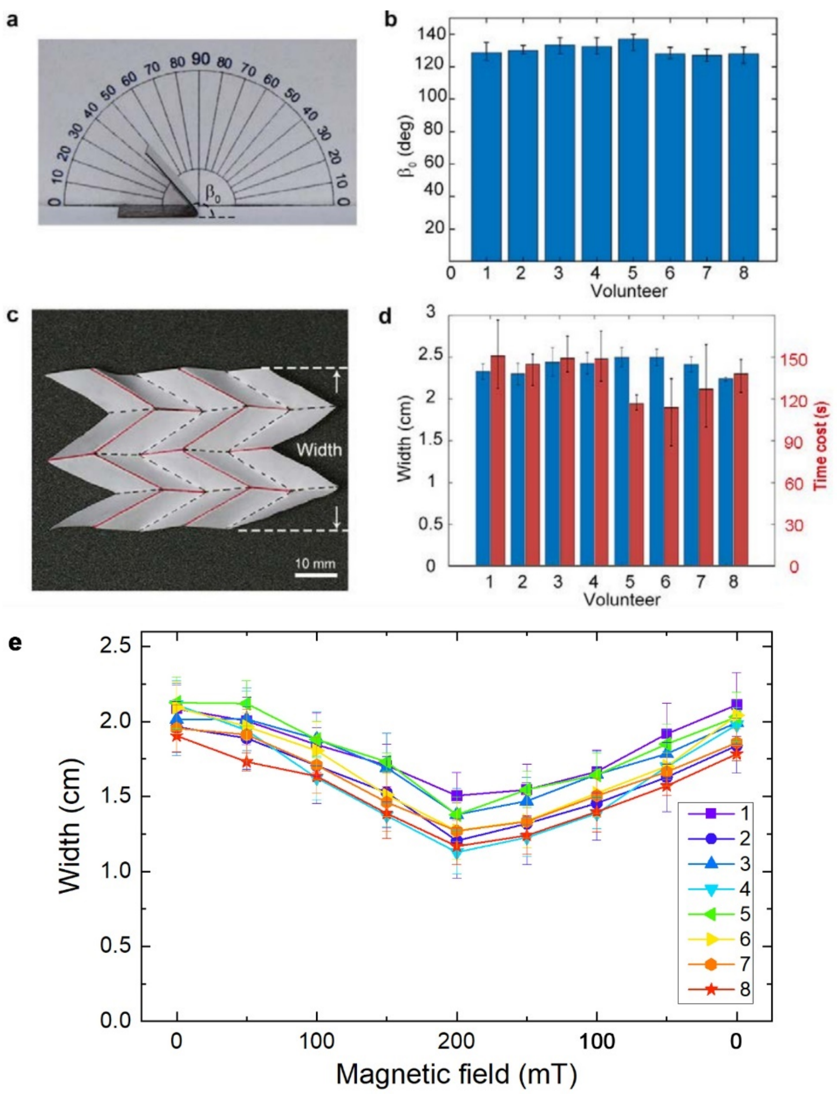

Supplementary Figure 11. Statistical studies of magneto-origami machines and performance evaluation by the robotic arm. (a) Optical image of a single-crease magneto-origami at the rest state. (b) The average and standard deviation of the rest angle of the single-crease magneto-origami is 130° and 4°, respectively. (c) Optical image of a Miura magneto-origami machine. (d) The average and standard deviation of the length of all the samples are approximately 2.4 mm and 0.14 mm, respectively. The average time cost is about 140 s. (e) The width of the Miura magneto-origami machine as a function of magnetic field strength. Each curve is the mean width of 7 samples by a specific volunteer. The error bar represents the width variation.

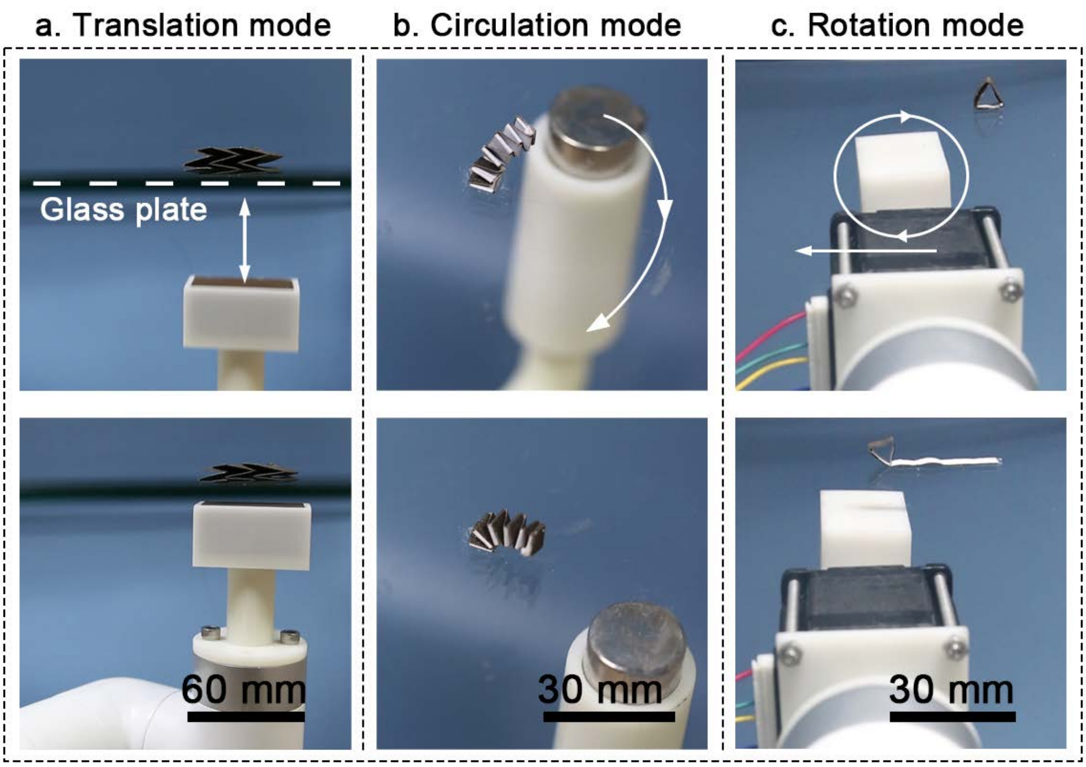

Supplementary Figure 12. Demonstration of three regular modes of manipulation by the robotic arm. (a) Translation mode. (b) Circulation mode. (c) Rotation mode.

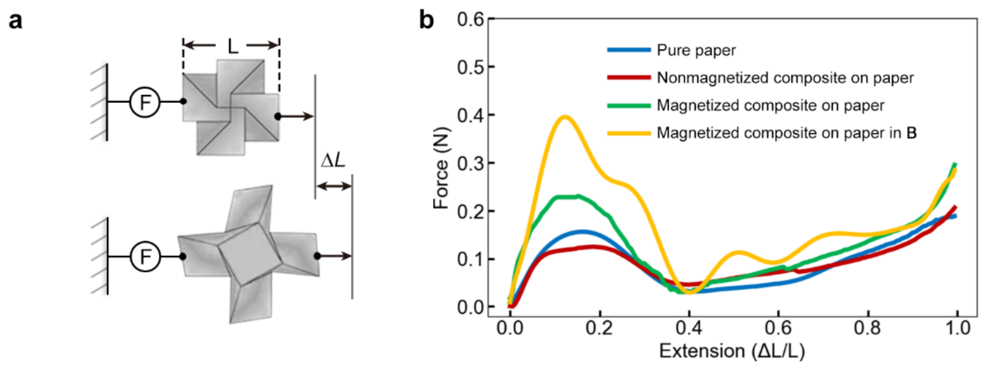

Supplementary Figure 13. Bistable behavior of the magneto-origami square-twist. (a) Schematic of the experimental setup to characterize the force (F)-extension (ΔL) relationship. (b) Tensile force as a function of normalized extension $\Delta L/L$ of magneto-origami square-twist made by (1) pure paper; (2) nonmagnetized composite on paper; (3) magnetized composite on paper; (4) magnetized composite on paper in magnetic field **B**.

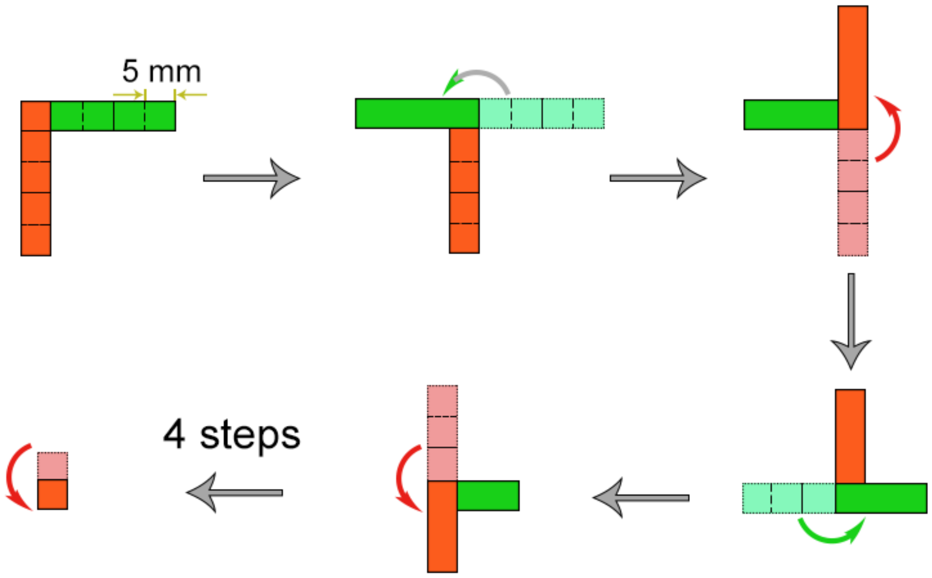

Supplementary Figure 14. Schematic illustration of the fabrication of magneto-origami spring actuator. First, the end sections of two magneto strips are glued together. Next, two magneto strips are alternatively folded. By repeating this, a magneto-origami spring actuator is eventually finished. The spring actuator was compressed tightly and magnetized by a strong pulsed magnetic field. When the compression was removed, the spring actuator returned to its rest state.

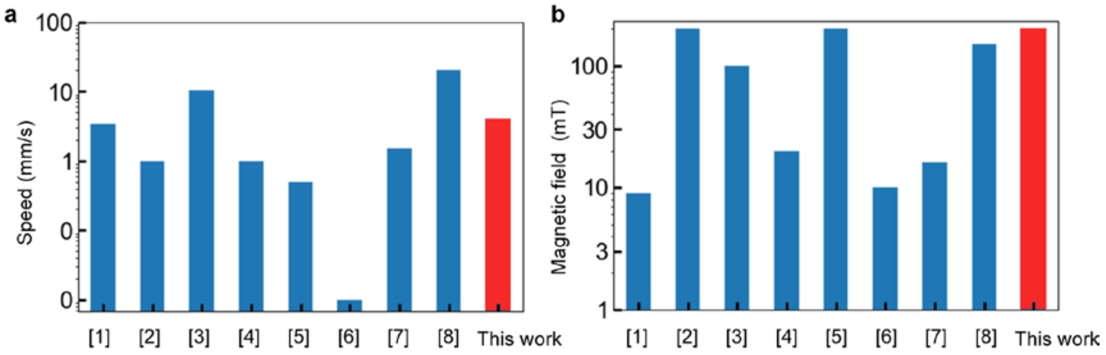

Reference
[1] T.Q. Xu, J.C. Zhang, M. Salehizadeh, O. Onaizah, E. Diller, Millimeter-scale flexible robots with programmable three-dimensional magnetization and motions, Sci Robot 4(29) (2019).
[2] X. Yang, W.F. Shang, H.J. Lu, Y.T. Liu, L. Yang, R. Tan, X.Y. Wu, Y.J. Shen, An agglutinate magnetic spray transforms inanimate objects into millirobots for biomedical applications, Sci Robot 5(48) (2020).
[3] Y. Li, Z.J. Qi, J.X. Yang, M.X. Zhou, X. Zhang, W. Ling, Y.Y. Zhang, Z.Y. Wu, H.J. Wang, B.A. Ning, H. Xu, W.X. Huo, X. Huang, Origami NdFeB Flexible Magnetic Membranes with Enhanced Magnetism and Programmable Sequences of Polarities, Adv Funct Mater 29(44) (2019).
[4] W.Q. Hu, G.Z. Lum, M. Mastrangeli, M. Sitti, Small-scale soft-bodied robot with multimodal locomotion, Nature 554(7690) (2018) 81-85.
[5] H.J. Lu, M. Zhang, Y.Y. Yang, Q. Huang, T. Fukuda, Z.K. Wang, Y.J. Shen, A bioinspired multilegged soft millirobot that functions in both dry and wet conditions, Nat Commun 9 (2018).
[6] H. Ceylan, N.O. Dogan, I.C. Yasa, M.N. Musaoglu, Z.U. Kulali, M. Sitti, 3D printed personalized magnetic micromachines from patient blood-derived biomaterials, Sci Adv 7(36) (2021).
[7] C. Li, G.C. Lau, H. Yuan, A. Aggarwal, V.L. Dominguez, S.P. Liu, H. Sai, L.C. Palmer, N.A. Sather, T.J. Pearson, D.E. Freedman, P.K. Amiri, M.O. de la Cruz, S.I. Stupp, Fast and programmable locomotion of hydrogel-metal hybrids under light and magnetic fields, Sci Robot 5(49) (2020).
[8] J.E. Park, J. Jeon, J.H. Cho, S. Won, H.J. Jin, K.H. Lee, J.J. Wie, Magnetomotility of untethered helical soft robots, Rsc Adv 9(20) (2019) 11272-11280.

Supplementary Figure 15. Comparisons to the relevant studies on the (a) moving speed and (b) required magnetic field of the spring actuator.

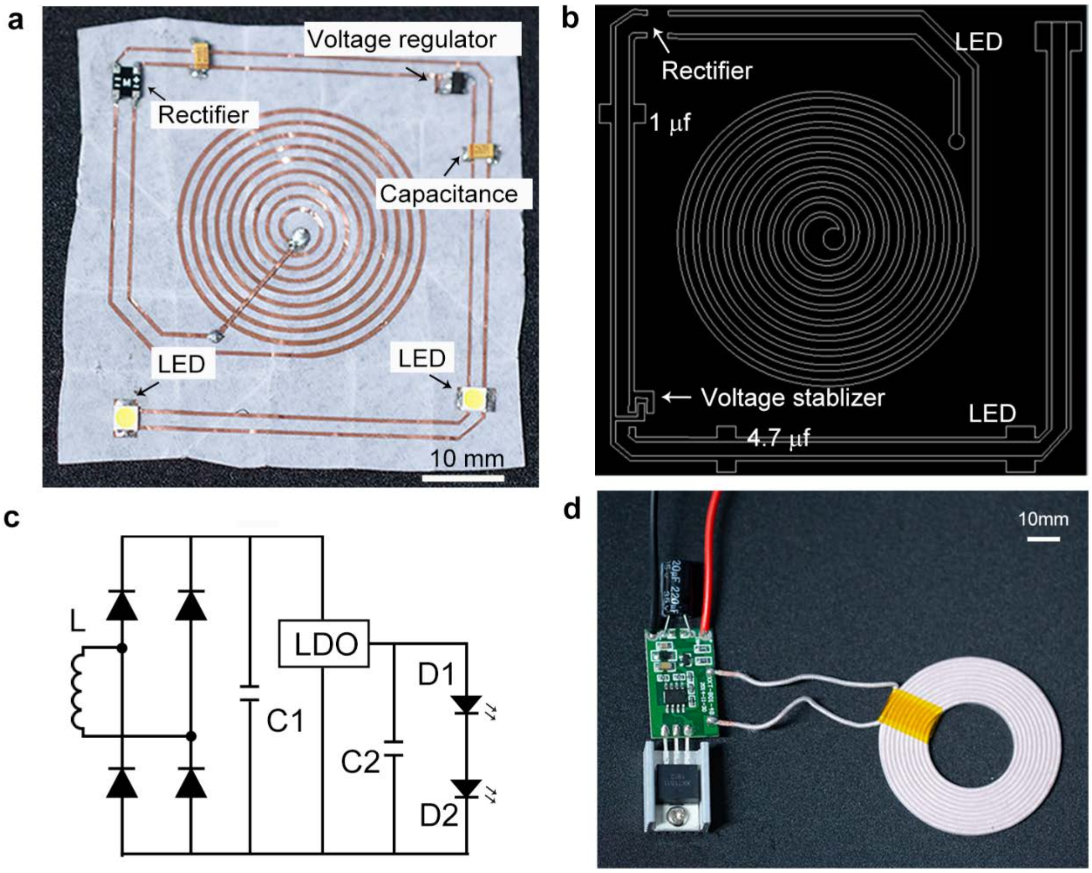

Supplementary Figure 16. Circuit design and wireless charging of MC robot. (a) The optical image of ME-robot with an electronic circuit on the paper layer. (b) The electronic circuit pattern was designed by AutoCAD software. The rectifier (LX10M, MBCN) is utilized to convert alternating current into direct current and the voltage regulator (LP2981IM5X-3.3) is employed to provide constant voltage output. (c) The schematic illustration of the wireless charging circuit of the ME-robot. (d) The optical image of the charging station. The external diameter and internal diameter of the induction coil of the charging station are 44 mm and 21 mm, respectively. The input voltage of the charging station was 25 V, and the direct distance between the charging station and ME-robot was approximately 6 mm.

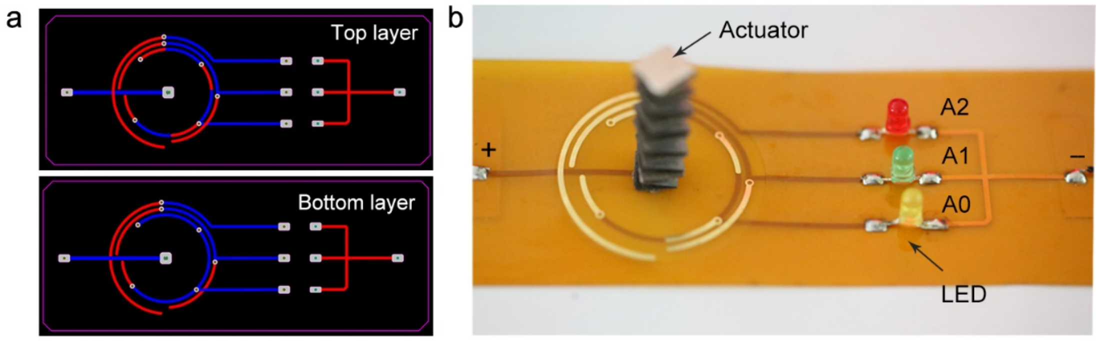

Supplementary Figure 17. The electronic design and demonstration of the magneto-origami 8-3 encoder. (a) The logical circuit design of 8-3 encoder. (b) Image of the 8-3 encoder. Red LED, green LED, and yellow LED represents the output A1, A2, and A0, respectively.

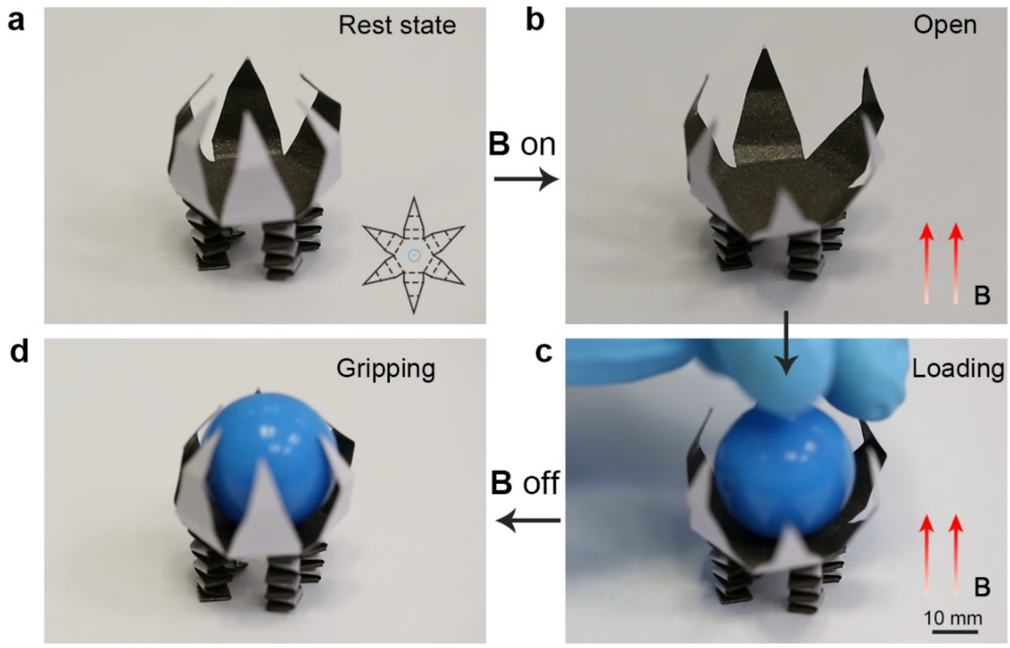

Supplementary Figure 18. The loading process of a sphere cargo on the magneto-origami quadruped robot. (a) Image of the magneto-origami quadruped robot. The insert image shows the folding and magnetization pattern of the gripper. (b) The gripper opens upon application of 150 mT actuating field, (c) sphere cargo is loaded in the gripper, and (d) the cargo was grasped by the gripper upon removal of the magnetic field. Scale bar: 5 mm.

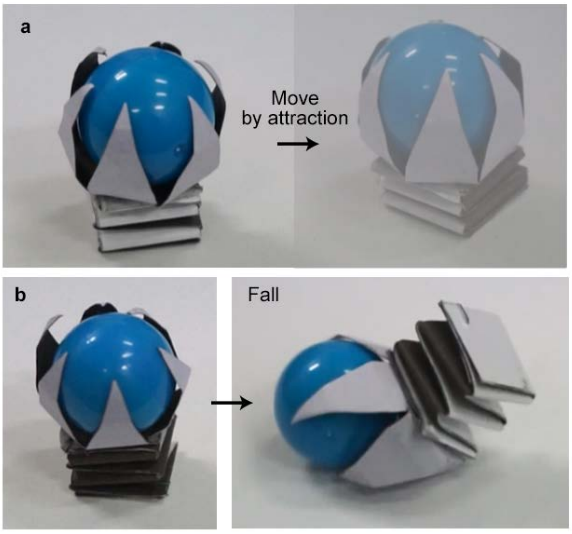

Supplementary Figure 19. Test of a single-legged robot. (a) A single-legged robot can carry the ball and move by magnetic attraction. (b) It falls when unloading the ball at the destination.

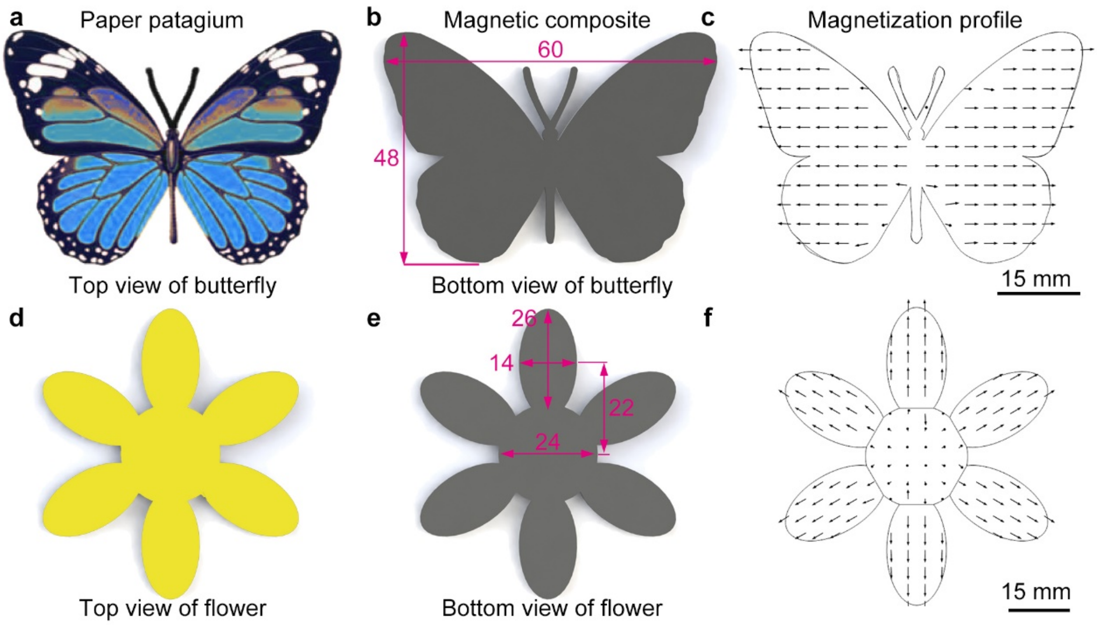

Supplementary Figure 20. Design of bionic magneto-origami machines. (a) Top view of the paper patagium of the bionic butterfly. (b) Bottom view of the magnetic composite of the bionic butterfly. (c) Magnetization profile of the bionic magneto-origami butterfly. (d) Top view of the paper patagium of the bionic flower. (b) Bottom view of the magnetic composite of the bionic flower. (c) Magnetization profile of the bionic magneto-origami flower.

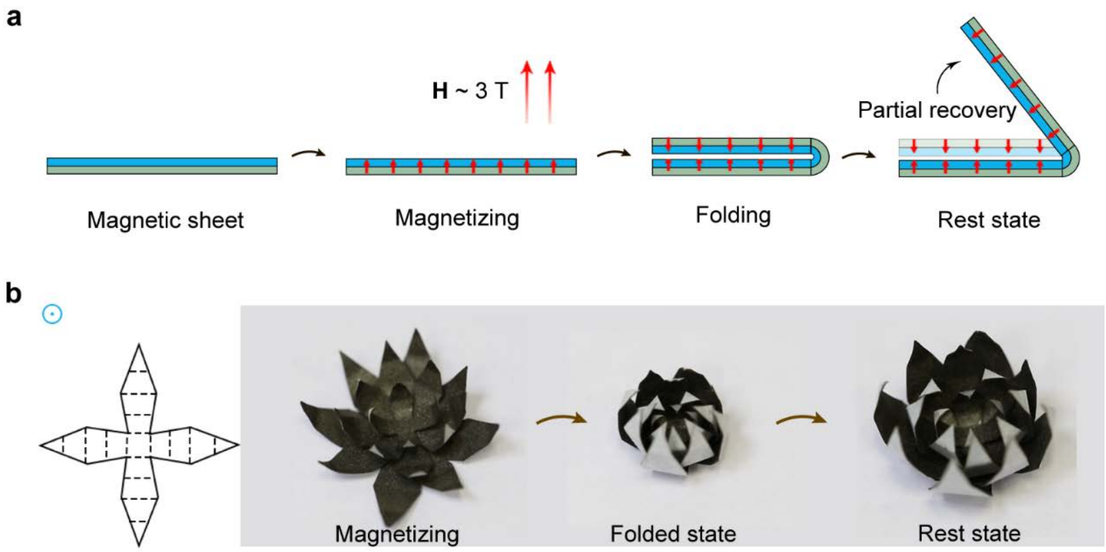

Supplementary Figure 21. Fabrication of magneto-origami machines by encoding the magnetization before folding. (a) Schematic illustration of encoding the magnetization before folding. (b) Example of a magneto-origami flower magnetized at the flat state and folded afterward.
