# Supplementary material for: High-throughput fabrication of soft magneto-origami machines
Source: Nat Commun. 2022 Jul 19;13:4177. doi: 10.1038/s41467-022-31900-5 (PMC9296529; doi:10.1038/s41467-022-31900-5)
Supplement: Supplementary file 3 — Description of Additional Supplementary Files [file 41467_2022_31900_MOESM3_ESM.pdf]

Description of Additional Supplementary Files

File Name: Supplementary Movie 1
Description: Roll to roll fabrication of magnetic sheet

File Name: Supplementary Movie 2
Description: Reversible folding and unfolding of magneto-origami machine

File Name: Supplementary Movie 3
Description: Fabrication and shape change of miura magneto-origami

File Name: Supplementary Movie 4
Description: Movement of controlling of the permanent magnets

File Name: Supplementary Movie 5
Description: Folding and unfolding of deployable machines

File Name: Supplementary Movie 6
Description: Sequential folding and unfolding of the magneto-origami strips

File Name: Supplementary Movie 7
Description: The magneto-origami spring actuator

File Name: Supplementary Movie 8
Description: Magneto-origami charging robot

File Name: Supplementary Movie 9
Description: Magneto-origami 8-3 encoder

File Name: Supplementary Movie 10
Description: Magneto-origami quadruped robot

File Name: Supplementary Movie 11
Description: Magneto-origami butterfly and flower

File Name: Supplementary Movie 12
Description: Magneto-origami flower
